# Epigenetics of the non-coding RNA *nc886* across blood, adipose tissue and skeletal muscle in offspring exposed to diabetes in pregnancy

**DOI:** 10.1186/s13148-024-01673-3

**Published:** 2024-05-07

**Authors:** Line Hjort, Sandra Stokholm Bredgaard, Eleonora Manitta, Irene Marques, Anja Elaine Sørensen, David Martino, Louise Groth Grunnet, Louise Kelstrup, Azadeh Houshmand-Oeregaard, Tine Dalsgaard Clausen, Elisabeth Reinhardt Mathiesen, Sjurdur Frodi Olsen, Richard Saffery, Romain Barrès, Peter Damm, Allan Arthur Vaag, Louise Torp Dalgaard

**Affiliations:** 1 Novo Nordisk Foundation Center for Basic Metabolic Research, Metabolic Epigenetics Group, Faculty of Health and Medical Sciences, University of Copenhagen, Copenhagen, Denmark; 2 Center for Pregnant Women With Diabetes, Department of Obstetrics, Rigshospitalet, Copenhagen, Denmark; 3 Department of Science and Environment, Roskilde University, Roskilde, Denmark; 4 Murdoch Children’s Research Institute, Parkville, Melbourne, VIC Australia; 5 Wal-Yan Respiratory Research Centre, Telethon Kids Institute, Perth Children’s Hospital, Nedlands, WA Australia; 6 Clinical Research, Steno Diabetes Center Copenhagen, Herlev Hospital, Herlev, Denmark; 7 Department of Clinical Medicine, University of Copenhagen, Copenhagen, Denmark; 8 Department of Gynecology and Obstetrics, Herlev Hospital, Herlev, Denmark; 9 Novo Nordisk A/S, Bagsværd, Denmark; 10 Department of Endocrinology, Rigshospitalet, Copenhagen, Denmark; 11 Centre for Fetal Programming, Statens Serum Institut, Copenhagen, Denmark; 12 Department of Paediatrics, University of Melbourne, Melbourne, VIC Australia; 13 Department of Clinical Sciences, Lund University, Malmö, Sweden

**Keywords:** Developmental programming, Intrauterine hyperglycemia, Gestational diabetes, Type 1 diabetes, Adipose, Muscle, ncRNA, nc886, DNA methylation, Gene expression, Epigenetics, VTRNA2-1

## Abstract

**Background:**

Diabetes in pregnancy is associated with increased risk of long-term metabolic disease in the offspring, potentially mediated by in utero epigenetic variation. Previously, we identified multiple differentially methylated single CpG sites in offspring of women with gestational diabetes mellitus (GDM), but whether stretches of differentially methylated regions (DMRs) can also be identified in adolescent GDM offspring is unknown. Here, we investigate which DNA regions in adolescent offspring are differentially methylated in blood by exposure to diabetes in pregnancy. The secondary aim was to characterize the RNA expression of the identified DMR, which contained the *nc886* non-coding RNA.

**Methods:**

To identify DMRs, we employed the bump hunter method in samples from young (9–16 yr, *n* = 92) offspring of women with GDM (O-GDM) and control offspring (*n* = 94). Validation by pyrosequencing was performed in an adult offspring cohort (age 28–33 years) consisting of O-GDM (*n* = 82), offspring exposed to maternal type 1 diabetes (O-T1D, *n* = 67) and control offspring (O-BP, *n* = 57). RNA-expression was measured using RT-qPCR in subcutaneous adipose tissue and skeletal muscle.

**Results:**

One significant DMR represented by 10 CpGs with a bimodal methylation pattern was identified, located in the *nc886*/*VTRNA2-1* non-coding RNA gene. Low methylation status across all CpGs of the *nc886* in the young offspring was associated with maternal GDM. While low methylation degree in adult offspring in blood, adipose tissue, and skeletal muscle was not associated with maternal GDM, adipose tissue *nc886* expression was increased in O-GDM compared to O-BP, but not in O-T1D. In addition, adipose tissue *nc886* expression levels were positively associated with maternal pre-pregnancy BMI (*p* = 0.006), but not with the offspring’s own adiposity.

**Conclusions:**

Our results highlight that *nc886* is a metastable epiallele, whose methylation in young offspring is negatively correlated with maternal obesity and GDM status. The physiological effect of *nc886* may be more important in adipose tissue than in skeletal muscle. Further research should aim to investigate how *nc886* regulation in adipose tissue by exposure to GDM may contribute to development of metabolic disease.

**Supplementary Information:**

The online version contains supplementary material available at 10.1186/s13148-024-01673-3.

## Background

Gestational diabetes mellitus (GDM) and obesity in pregnancy are associated with an increased risk of long-term metabolic disease in the offspring [1]. Growing evidence suggests that epigenetic variation established in utero links the adverse intrauterine environment of GDM to the increased metabolic risks [2, 3]. However, the exact mechanisms leading to metabolic phenotypes later in life remain unclear.

Elucidating the role of epigenetic variation in human disease is complicated by many factors, including cell type/tissue specificity and variation over time. Despite this, some epigenetic marks are established very early in development and are maintained across tissue types over time. Metastable epialleles are defined as genomic regions of DNA methylation established by apparently stochastic events in early fetal life, resulting in uniform cross-tissue epigenetic patterns [4]. However, maternal nutrition around the time of conception has been demonstrated to affect DNA methylation at metastable epialleles [5]. Whether these are modifiable or change over time in response to the environment is, in general, not known.

We previously performed an array-based Epigenome Wide Association Study (EWAS) between preadolescent and adolescent GDM offspring and controls, and identified differentially methylated probes associated with exposure to GDM or high maternal pre-pregnancy BMI, or with both of these maternal factors [6]. However, at present, the extent to which these factors impact differentially methylated regions (DMRs), including metastable epialleles, remains unclear, as is the relationship to cardio-metabolic health of the offspring. With growing awareness of the limiting factors concerning the statistical power when only focusing on single differentially methylated CpG sites, we applied the Bump Hunting method [7] on the same dataset, to explore if DMRs were present in GDM offspring. This method allows the identification of regions of multiple CpG sites regulated together.

The overall aim of the present study was therefore to identify DMRs in blood from young GDM exposed offspring compared to controls: One DMR was identified, covering the small non-coding RNA *nc886*. The *nc886* locus is epigenetically interesting as it is maternally imprinted in approximately 75% of offspring, and further controlled by both a common SNP located in the vicinity [8] and by environmental exposures: It was previously found that the likelihood of *nc886* DMR being hypomethylated in blood and hair follicles was increased, when conception occurred during the dry season compared to during the rainy season [4]. Those results indicated that the environmental effect on the *nc886* DMR occurs in the early embryo and that *nc886* is likely to hold capacity as a metastable epiallele, responsive to the fetal nutritional state [4]. The secondary aim was to characterize DNA methylation and RNA expression of the identified DMRs and *nc886* in blood, skeletal muscle and adipose tissue in a separate, independent cohort of adult offspring exposed to diabetes and/or obesity in pregnancy.

## Methods

### Clinical study cohorts

#### Young offspring cohort

A GDM subgroup (608 children), together with a randomly selected control group (626 children) was established from the Danish National Birth Cohort in 2012–14, when the children were aged 9–16 years (6, 9–11). In brief, the offspring underwent an examination including anthropometric measurements and fasting blood samples for measurement of glucose, hsCRP, insulin, C-peptide and lipid profiles. All samples were analyzed in agreement with standard laboratory procedures. For DNA sampling, EDTA tubes (10 ml, BD) were placed directly on ice after collection of venous blood and processed within 10 min. Buffy coat was prepared from EDTA tubes and stored at − 80 °C.

#### The adult offspring cohort

The CoCo offspring cohort (Copenhagen Offspring Cohort, Rigshospitalet) comprises 206 adult offspring born at Rigshospitalet, Copenhagen, Denmark, between 1978 and 1985, of women with either diet-treated GDM (O-GDM) in pregnancy, T1D in pregnancy (O-T1D), and a random control group of offspring from normoglycemic pregnancies representing the background population (O-BP) [12]. In 2012, the offspring were invited to a clinical follow-up visit, which included anthropometrics and DEXA scans, fasting blood samples, an oral glucose tolerance test (OGTT), and measurement of glucose, hsCRP, HbA_1c_, insulin, C-peptide and lipid profiles [13]. All samples were analyzed using standard laboratory procedures. Biopsies of abdominal subcutaneous adipose tissue (SAT) and vastus lateralis skeletal muscle were obtained after the overnight fast, frozen in liquid nitrogen, and stored at − 80 °C. Buffy coat was prepared from EDTA tubes and stored at − 80 °C. For RNA sampling, venous blood was collected in PAXgene tubes (2.5 mL, PreAnalytiX), according to the manufacturer’s directions and stored at − 80 °C.

The study designs and protocols were approved by the Regional Scientific Ethical Committee of the municipalities of Copenhagen and Frederiksberg (and conformed to the Helsinki II declaration).

### DNA methylation analysis

#### DNA extraction

Genomic DNA was extracted from buffy coat using the QIAamp 96 DNA blood kit, from muscle biopsies using the DNeasy blood and tissue kit, and from SAT biopsies using the QIAamp DNA mini kit (Qiagen, Valencia, CA, USA). Bisulfite conversion of blood DNA was performed with 500 ng DNA using the EZ-96 DNA Methylation Kit (ZYMO Research). Bisulfite conversion was performed with 400 ng DNA (SAT) and 500 ng DNA (muscle) using the Epitect Bisulfite Kit (Qiagen).

#### Epigenome-wide microarray study

DNA methylation profiling was performed using the Infinium HumanMethylation450 BeadChip (Illumina) on 93 GDM and 95 control offspring from the young cohort as previously published [6]. In brief, raw *β*-values were preprocessed using the Minfi package from the Bioconductor project [14] and quality control assessment performed including testing for cell type composition differences using surrogate variable analysis [15, 16]. For identification of differentially methylated CpG regions (DMRs) between GDM and control offspring, we used the bump hunter function (Bioconductor version 3.16) identifying and ranking the most DMRs across the genome, based on smoothing of the differential methylation signal [7, 14]. Bump hunter was chosen as the most suitable tool for analysis of the Infinium HumanMethylation450 BeadChip platform, for which it has been widely used [17–19] Methylation percentages were estimated as *β*-values given by *β* = Meth/(Unmeth + Meth + 100) to facilitate biological interpretation.

#### Specific DNA methylation analysis using pyrosequencing

**Fig. 1:**
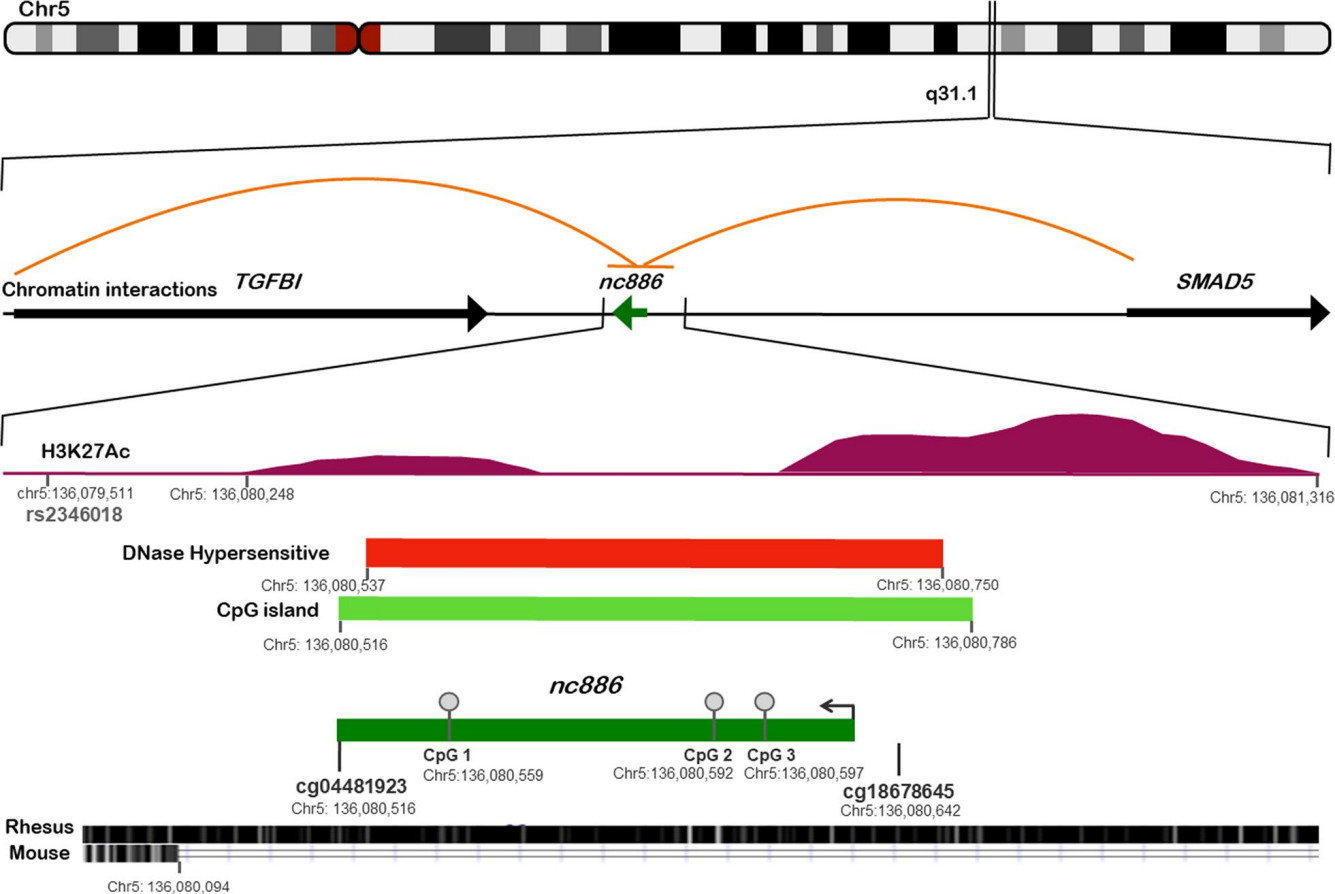
Overview of the genomic region of *nc886* and the specific CpGs sites analyzed in the pyrosequencing assay (CpG 1 (Chr5:136,080,559), CpG 2 (Chr5:136,080,592) and CpG 3 (Chr5:136,080,597) in relation to the genomic structure of the nc886 region. The non-coding RNA nc886 is located on Chr. 5q31.1 between TGFBI and SMAD5 and is co-localized with a CpG island, which forms the DMR. The region forms open, DNase-sensitive chromatin, which share regulatory regions with the other genes in the region (indicated by chromatin interactions to TGFBI and SMAD5 and H3K27Ac ChIP-seq). The nc886 is restricted to primates and is not conserved in other mammalian species. Source of tracks: GenomeBrowser (genome.uscs.edu), Encode regulation: Subtrack DNase HS (skeletal muscle, skeletal muscle myoblast, skeletal muscle myotube), subtrack Layered H3K27Ac (on 7 cell lines from ENCODE), subtrack in situHiC/microC (on H1-hESC and HFFc6). Conservation: Subtracks rhesus, mouse. Nucleotide positions given are for hg38

Measurement of specific *nc886* CpG DNA methylation levels in the adult cohort was taken using pyrosequencing (PyroMark Q48 Autoprep, Qiagen,) with PyroMark Q48 reagents. PCR and sequencing primers were designed using PyroMark Assay Design 2.0 (Additional file 1: Table S1) and covered three CpGs located between the cg04481923 and cg18678645 CpGs from the beadchip array (Fig. 1). The localizations of the three CpG sites were: CpG 1 (Chr5:136,080,559), CpG 2 (Chr5:136,080,592) and CpG 3 (Chr5:136,080,597). Specificity was examined by running the PCR products on a gel, securing that there was only one specific band, and estimating it to have the correct length according to the design of the pyrosequencing PCR product.

### Genotyping of rs2346018 C/A single nucleotide polymorphism (SNP)

For 190 (74 O-GDM, 64 O-T1D, 52 O-BP) of the 206 adult offspring participants, genotyping data from peripheral blood buffy coat DNA collected during the first follow-up study [12] were available from a previously conducted genome-wide association study (GWAS) meta-analysis study [20]. Blood DNA SNPs were genotyped using the Human CoreExome-24v1.0 platform, and the SNPTEST v2.5.2 software was used for interpretation of the results. Genotype imputation was conducted for all autosomes on build 37 (Hg19) on the forward strand, and imputed to the Human Reference Consortium (HRC) reference panel. The imputed rs2346018 C/A SNP, located on chromosome 5, position 135415300 (hg37)/136079511(hg38), were extracted from the genotyping dataset to be included in the present study.

### Gene expression analysis using qPCR

MiRNeasy Mini Kit (Qiagen) was used for total RNA extraction from muscle and SAT. QuantiTect Reverse Transcription Kit was used for cDNA synthesis (Qiagen). In total, cDNA from 168 SAT samples, 187 muscle samples and 200 blood samples were available and analyzed using qPCR. Primers for nc886 were designed using NCBI Primer-BLAST tool (Additional file 1: Table S1). The expression of nc886 was measured in all samples with Transcription factor II B (*TFIIB*) (in adipose tissue) and Hypoxanthine–guanine Phosphoribosyl Transferase (*HPRT*) (in skeletal muscle) as endogenous control genes using QuantiTect SYBR® Green PCR Kit (Qiagen). Both genes have been evaluated previously as suitable references in these tissues [21, 22]. The abundance of *nc886* in blood buffy coat was too low for reliable quantification, an observation supported by publically available small RNA-seq data from peripheral blood mononuclear cells (FANTOM5 consortium, SlideBase [23]. The relative gene expression was quantified using the standard curve principle. Each sample was performed in duplicates using the Viia 7 Real-Time PCR System.

### Statistical analyses

Normally distributed data are presented as mean ± SD and compared by Students *T*-test. Non-normally distributed data are presented as median and interquartile range (IQR) and compared by the Mann–Whitney *U* test. Proportions of categorical data were tested using Fishers exact test. Correlation analyses were made with Pearson’s and Spearman’s Rank correlation test. Hierarchical clustering analysis was performed within the “hclust” function in the “dplyr” package, using R version 4.2.1. Multivariate linear regression models were applied to address potential confounding effects on the association between exposure and offspring DNA methylation/gene expression. We tested whether the potential confounding covariates including maternal pre-pregnancy BMI, offspring/maternal age, mode of delivery, gestational age at birth, offspring waist-hip circumference, body fat percentage and muscle mass were associated with methylation status of nc886. In final models only maternal pre-pregnancy BMI and offspring sex and body fat percentage were included, besides nc886 genotype. Differences were estimated as β-coefficients and 95% CI. Assumptions of equal variance and normally distributed residuals were visualized in QQ plots and histograms. A binary logistic model was used to assess the risk associated with imprinting given maternal GDM, using the LOGISTIC procedure in R, maternal GDM status as binary classifier and gene expression of *nc886* as explanatory variable. All statistical analyses were performed using the SAS 9.4, RStudio 4.1.0/R 4.2.1, and GraphPad Prism version 9.0. A *p* ≤ 0.05 was considered statistically significant. When performing multiple tests by examining each individual CpG site, in each tissue, we corrected for multiple testing by Bonferroni correction, and hence considered a *p*-value statistically significant, when *p* ≤ 0.00556 (0.05/nine tests).

## Results

### Clinical characteristics of the offspring cohorts

We investigated two cohorts of offspring of GDM mothers: A young cohort established as part of the National Danish Birth Cohort and an adult cohort, the Copenhagen Offspring Cohort (CoCo) comprising adult offspring of women with either diet-treated GDM (O-GDM) in pregnancy, T1D in pregnancy (O-T1D), and a control group of offspring from normoglycemic pregnancies representing the background population (O-BP). Both the young and the adult O-GDM had mothers with a ~ 3.1 kg/m^2^ higher pre-pregnancy BMI, compared to the controls (*p* < *0.0001*), although the mothers of the young offspring in general had higher pre-pregnancy BMI of ~ 2.0 kg/m^2^ compared to mothers of the adult offspring (Table 1) [10, 13]. Among young offspring, BMI, C-peptide and insulin levels were significantly higher, or tended to be higher, in O-GDM compared to O-BP. Among adult offspring, the O-GDM and O-T1D in general showed similar clinical characteristics compared to the O-BP; however, O-GDM and O-T1D had higher glucose levels during the OGTT compared to O-BP (Table 1).

**Table 1:** Clinical characterization of the offspring cohorts

Young offspring cohort (age 9–16 years)			
	O-GDM	O-BP	*p* value
	*n* = 92	*n* = 94	
Maternal pre-pregnancy BMI (kg/m^2^)	26.4 (± 4.8)	23.4 (± 4.7)	** < *0.0001***
Age (years)	11.7 (± 1.6)	11.7 (± 1.5)	*0.79*
Sex (female)	49%	50%	*0.68*
BMI (kg/m^2^)	19.1 (3.42)	17.8 (2.48)	***0.01***
Total body fat (%)	30.0 (± 9.2)	27.2 (± 6.2)	*0.12*
Fasting insulin (pmol/l)	81.4 (48.3)	67.2 (32.4)	*0.06*
Fasting C-peptide (pmol/l)	616 (± 235)	546 (± 174)	***0.02***
Fasting glucose (mmol/l)	4.94 (0.50)	4.92 (0.48)	*0.90*

Adult offspring cohort (age 28–33 years)					
	O-GDM *n* = 82	O-T1D *n* = 67	O-BP *n* = 57	*p* value	
				O-GDM versus O-BP	O-T1D versus O-BP
Maternal pre-pregnancy BMI (kg/m^2^)	24.3 (± 5.6)	21.7 (± 1.9)	21.2 (± 3.5)	** < *0.0001***	*0.30*
Age (years)	30.8 (± 2.1)	31.3 (± 2.4)	31.3 (± 2.4)	*0.27*	*0.83*
Sex (female)	48%	54%	54%	*0.43*	*0.94*
BMI (kg/m^2^)	24.7 (21.8–27.1)	24.2 (22.1–27.7)	24.2 (21.7–26.6)	*0.71*	*0.46*
Total body fat (%)	31.2 (± 9.1)	32.5 (± 9.8)	29.8 (± 7.9)	*0.35*	*0.09*
Fasting insulin (pmol/l)	48.3 (32.3–73.5)	50.2 (40.4–69.5)	47.8 (35.6–70.2)	*0.83*	*0.27*
Fasting C-peptide (pmol/l)	662 (± 250)	683 (± 147)	616 (± 197)	*0.23*	*0.10*
Fasting glucose (mmol/L)	4.9 (4.5–5.1)	4.9 (4.7–5.2)	4.9 (4.6–5.1)	*0.90*	*0.59*
2 h glucose (mmol/L)	6.0 (± 1.81)	6.3 (± 1.69)	5.3 (± 1.23)	***0.02***	***0.001***
Total cholesterol (mmol/L)	4.73 (± 0.73)	4.76 (± 0.74)	4.86 (± 0.88)	*0.38*	*0.51*
LDL (mmol/L)	2.80 (± 0.66)	2.73 (± 0.68)	2.89 (± 0.77)	*0.49*	*0.22*
HDL (mmol/L)	1.37 (± 0.35)	1.48 (± 0.33)	1.42 (± 0.41)	*0.46*	*0.40*

Data are presented as mean (±SD), median (IQR), or percentage

*O-GDM* offspring of mothers with GDM, *O-T1D* offspring of mothers with T1D, *O-BP* offspring of mothers from the background population, *mBMI* Maternal BMI.

The differences between O-GDM versus O-BP or O-T1D versus O-BP for the continuous variables with parametric and nonparametric distributions were tested by independent samples *t-*test or Mann–Whitney *U* test, respectively. For the categorical variable (sex) the distribution differences were assessed by the χ^2^ test. *p* values < 0.05 are in bold

### Blood *nc866* DNA methylation in young offspring

**Fig. 2:**
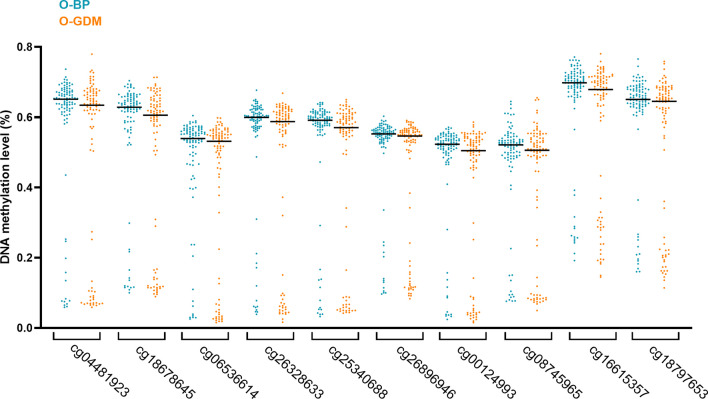
Young offspring DNA methylation data shown with medians from the array study of the *nc886* DMR region from the young cohort (control offspring (O-BP) versus GDM offspring (O-GDM)). Displayed are individual data points as well as median (black horizontal lines)

Using the bump hunting algorithm, we identified one significantly differentially methylated region, a > 600 bp long DMR including a 271 bp long CpG island consisting of 24 CpGs, of which 10 sites were measured on the array (Additional file 1: Table S2). Only one gene is located within this DMR, the non-coding vault RNA *nc886* (Fig. 1), also known as *vtRNA2-1*. This DMR exhibited a pattern of clearly bimodal methylation levels, where the DNA across all ten sites, was either hypo-methylated (having less than 30% methylation) or hemi-methylated (having approximately 50–80% methylation) in each individual, corresponding to a median methylation percentage across the DMR ranging between 11 and 60% methylation (Fig. 2). Because the *nc886* DMR displayed a biomodal methylation pattern, we conducted an objective estimation of whether each participant was in the hypo- or hemi-methylated group, by performing an unsupervised hierarchical cluster analysis, combing all data from the 10 CpGs of the DMR. The cluster analysis (Additional file 1: Fig. S1) showed that participants fell into two distinct groups, whereof 37 (20%) participants were in the hypo-methylated (suggested bi-allelic hypomethylation) and 148 (80%) in the hemi-methylated (suggested mono-allelic hypomethylation) group. O-GDM participants had ~ 10% lower average methylation compared to O-BP across all CpGs within the DMR (Fig. 2), resulting from the significantly higher frequency of O-GDM than O-BP, in the hypo-methylated group (Fishers exact test, *p* = 0.04, Table 2).

**Table 2:** Frequencies of offspring in *nc886* hypo-methylated or hemi-methylated groups

	*nc886* group		
	Hypo-methylated	Hemi-methylated	*p* value
Young offspring			
O-GDM	24 (27%)	66 (73%)	
O-Control	13 (14%)	82 (86%)	
In total	37 (20%)	148 (80%)	***p* = *0.04***
Adult offspring			
O-GDM	16 (22%)	57 (78%)	
O-BP	14 (27%)	38 (73%)	
O-T1D	10 (17%)	50 (83%)	
In total	40 (22%)	145 (78%)	*p* = *0.42*

Proportions of categorical data are shown as *n* (%) and were tested using Fishers exact test

### *Nc886* methylation across tissues of adult offspring

**Fig. 3:**
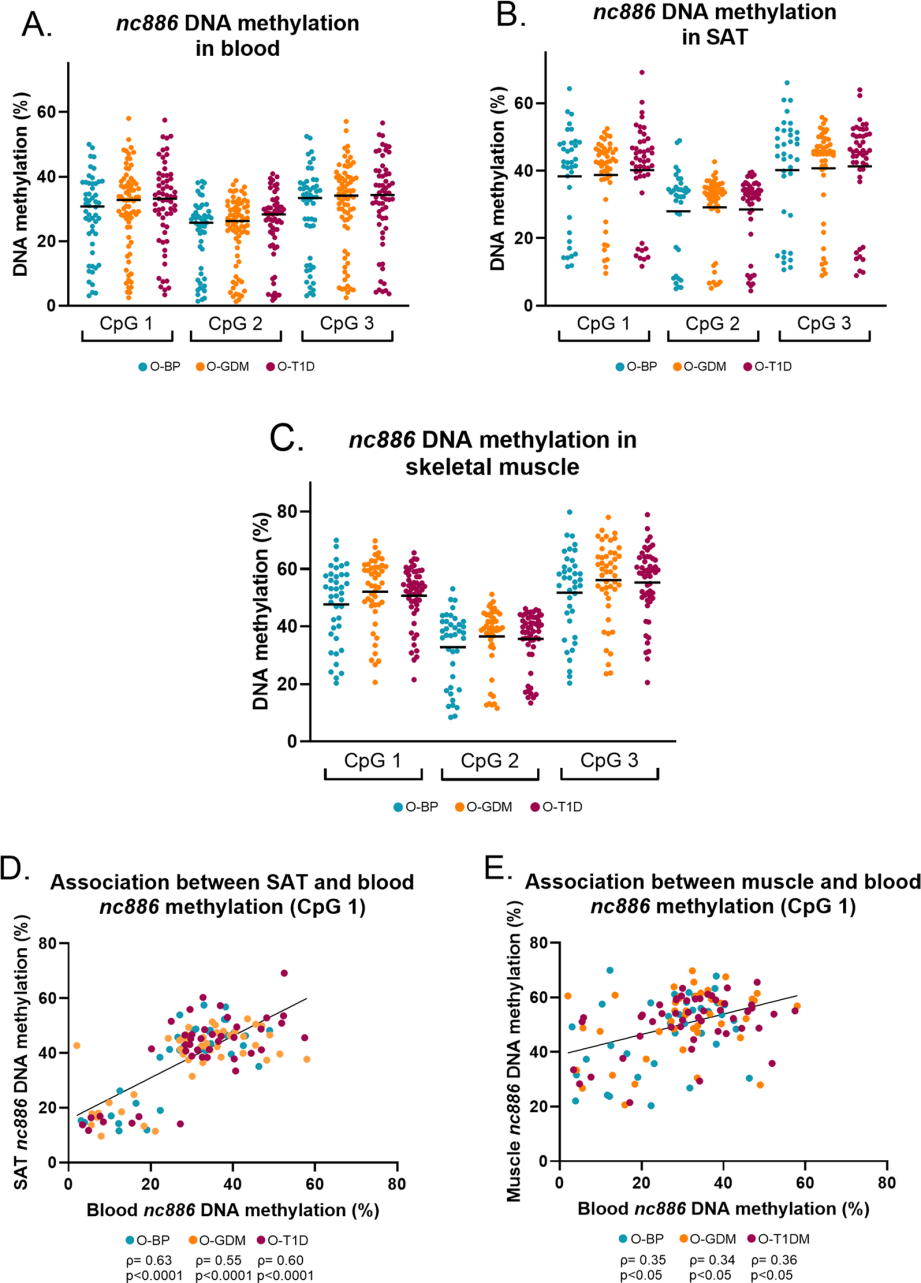
Adult offspring DNA methylation profiles of the *nc886* region covering three CpGs with pyrosequencing analysis, across the different adult offspring groups in **A** blood, **B** subcutaneous adipose tissue (SAT), and **C** skeletal muscle tissue. Data are presented with median (black horizontal lines) and statistical differences between expression levels were determined by the Mann–Whitney U test for nonparametric data (all non-significant). Spearman rank correlation models of DNA methylation levels between **D** subcutaneous adipose tissue (SAT) and blood, and **E** skeletal muscle tissue and blood

In adult offspring tissues, we also detected bimodal methylation patterns, however, with a less pronounced hypo- or hemi-methylation pattern compared to the younger offspring by HM450K array analysis (Fig. 3A–C). Clustering revealed that 40 offspring (22%) were in the hypo-methylated group, and 166 (78%) in the hemi-methylated group (Additional file 1: Fig. S2), however there were no difference in proportion of offspring from the different exposure groups between the hypo- and hemi-methylated groups (Fishers exact test, *p* = 0.42, Table 2).

We did not observe any differences between *nc886* DNA methylation levels of O-GDM or O-T1D compared to the O-BP, in any of the three tissues (Fig. 3A–C). This result did not change when taking into account the covariates: Diabetes in pregnancy, pre-pregnancy BMI and offspring nc886 genotype and sex (Additional file 1: Table S3). Median methylation percentages were different across the three tissues for the three CpG sites included in the assay, ranging between 27 and 58%, with methylation being lowest in blood cells and highest in muscle tissue. Moreover, for each type the degree of methylation was positively correlated intra-individually (Additional file 1: Table S4, Fig. 3D–E).

### An nc886 C/A SNP is not associated with nc886 DNA methylation degree

Allele-specific DNA methylation can be associated with genetic polymorphisms [8]. The nc886 DMR contains a CTCF binding site with a A/C SNP, rs2346018, which disrupts the CTCF motif on the centromeric side of the DMR. This SNP was previously examined for potential causality regarding association with *nc886* DNA methylation degree, with inconsistent conclusions regarding impact on DNA methylation [8, 24]. For the follow-up cohort of adult offspring rs2346018 genotype data were available, and we did not observe any association between offspring groups status (O-BP, O-GDM, O-T1D) and rs2346018 genotype (Fisher’s exact test, *p* = 0.68, Supp. Table 5, neither did we find that the C vs. A allele of this SNP was associated with *nc886* epiallele methylation status defined as hypo- versus hemi-methylated (Fisher’s exact test, *p* = 0.17, Supp. Table 5).

### *Nc886* RNA expression in adipose and muscle tissue from adult offspring of women with GDM or T1D in pregnancy

**Fig. 4:**
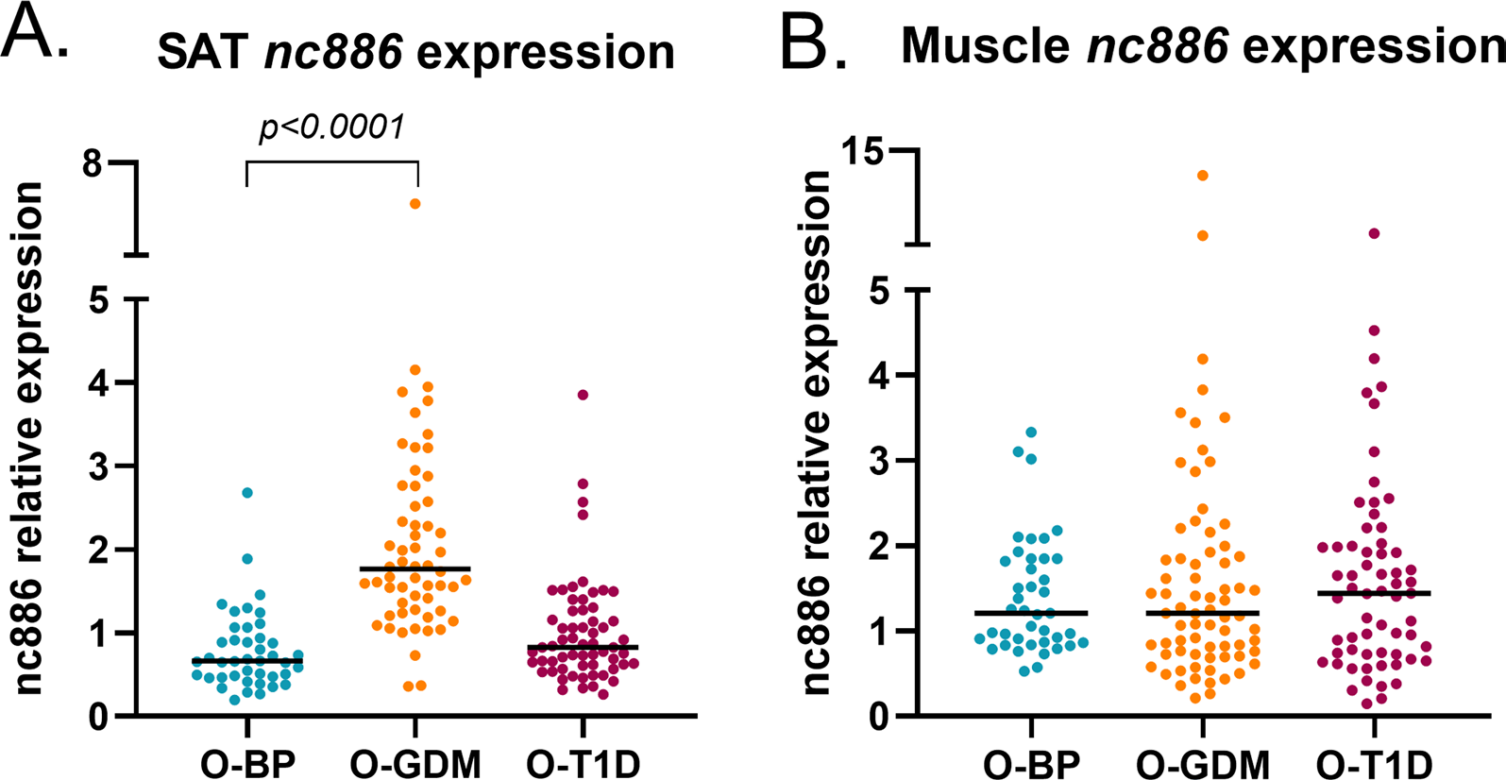
Adult offspring relative expression of full-length *nc886* in **A** adipose tissue and **B** skeletal muscle across the three offspring groups. Expression levels were normalized to *TFIIB* for adipose tissue and *HPRT* for skeletal muscle. Data are presented with median and statistical differences between expression levels were determined by the Mann–Whitney *U* test for nonparametric data

To investigate if DNA methylati6n degree of the *nc886* region is associated with *nc886* RNA expression, we assessed *nc886* RNA levels in the adult cohort in skeletal muscle and subcutaneous adipose tissue (SAT). We observed significant negative correlations between *nc886* methylation and expression in both tissues, with the strongest correlation in adipose tissue (Additional file 1: Table S6). In SAT, *nc886* RNA expression levels were significantly higher in the O-GDM compared with the O-BP in both group-wise comparisons (Fig. 4A, GDM: 1.8 (1.3,2.8) vs O-BP: 0.7(0.5,0.9), *p* < *0.0001*), and in multiple regression analysis, adjusting for maternal pre-pregnancy BMI, offspring nc886 SNP genotype and sex (Table 3, model 1, *p* = *0.007*). Importantly, adjusting for offspring body fat percentage did not affect the results (Table 3, model 2 *p* = *0.005*). Moreover, both without (model 1) and with (model 2) adjustment for offspring body fat percentage, maternal prepregnancy BMI was positively associated with SAT *nc886* expression (Table 4, *p* = *0.03, p* = *0.02*). We further tested the association between maternal GDM status and gene-expression levels of *nc886* using logistic regression: Maternal GDM resulted in an odds-ratio of 25.6 (95% CI: 6.0–101.0, logistic regression), *p* < 0.0001, for offspring having increased SAT *nc886* RNA expression, with concordance estimates of 92.7% indicating that almost all mother–offspring pairs display this association of maternal GDM to increased SAT *nc886* levels (Additional file 1: Table S7), indicating that the higher the level of gene expression of *nc886* the more likely it is that the mother had GDM during pregnancy. In contrast, the SAT *nc886* expression level in O-T1D was not altered compared to O-BP (Fig. 4A) and in skeletal muscle, no differences in *nc886* expression were found in either of the offspring groups (Fig. 4B).

**Table 3:** Association between adult offspring *nc886* expression and maternal diabetes and pre-pregnancy BMI

Outcomes	Association to group compared to O-BP				Association to maternal pre-pregnancy BMI	
	O-GDM		O-T1D			
	*β* (95% CI)	*p* value	*β* (95% CI)	*p* value	*β* (95% CI)	*p* value
Model 1 (adjusted for maternal diabetes status, maternal pre-pregnancy BMI as continuous trait, offspring *nc886* genotype, and sex)						
* SAT nc886*	1.28 (0.36, 2.20)	***0.007***	0.22 (− 0.03, 0.48)	*0.08*	0.08 (0.01, 0.15)	***0.03***
* Muscle nc886*	0.36 (− 1.86, 2.58)	*0.75*	− 0.15 (− 0.93, 0.63)	*0.70*	0.09 (− 0.08, 0.25)	*0.31*
Model 2 (model 1 + adjusted for offspring total body fat percentage)						
* SAT nc886*	1.33 (0.41, 2.25)	***0.005***	0.20 (− 0.05, 0.45)	*0.12*	0.09 (0.02, 0.17)	***0.02***
* Muscle nc886*	0.37 (− 1.88, 2.62)	*0.74*	− 0.19 (− 0.98, 0.61)	*0.64*	0.06 (− 0.12, 0.23)	*0.54*

Estimated differences in relative gene expression degree between exposed groups compared to O-BP analyzed using a general linear model. Data are presented as β (95% CI) and *p* value*.* β-estimate shown as difference in *nc886* expression relative to the reference gene.

*O-BP* offspring of mothers from the background population, *O-GDM* offspring of mothers with gestational diabetes, *O-T1D* offspring of mothers with type 1 diabetes, *SAT* subcutaneous adipose tissue, *mBMI* maternal BMI

### Association of *nc886* SAT expression profiles with clinical parameters

As previously mentioned, an association between peripheral blood *nc886* DNA methylation and gene expression levels and glucose and insulin plasma levels was recently described [25]. Therefore, we examined the association between SAT *nc886* DNA methylation and gene expression levels and metabolic traits in the adult offspring. In simple, unadjusted correlation analysis, combining all three groups to increase sample size and statistical power, *nc886* expression in SAT was positively correlated with maternal pre-pregnancy BMI (*p* = 0.006), but was not correlated with the offspring’s own total body fat percentage (Additional file 1: Fig. S2, Table S8), which is in line with the results observed in Table 3, based on the multiple regression analysis. Furthermore, we found that SAT *nc886* expression was positively correlated with offspring fasting insulin (*r* = 0.18, *p* = 0.026) and C-peptide (*r* = 0.18, *p* = 0.025), and negatively correlated with HDL cholesterol (*r* = − 0.24, *p* = 0.002) (Additional file 1: Fig. S2B–D, Table S8) indicating that a more cardiometabolically challenged phenotype could be associated with increased SAT levels of *nc886*, although with modest correlation values*.*

For comparison with publically available methylation databases, we queried the EWAS catalogue (https://ewascatalog.org/), the ARIES (http://www.mqtldb.org/) and HELIX (https://helixomics.isglobal.org/) databases. In brief, data lookups indicated that the degree of methylation increases as a function of age in some models (though not all), and that the expression of the DNA repair gene DNAJB6 is positively correlated with methylation at the nc886 DMR, in samples from children. However, no statistically significant associations with DNA methylation at this locus were identified in response to pregnancy or childhood environmental exposures (tobacco smoking, environmental contaminants or air quality), based on EWAS catalogue data (Additional file 1: Table S9).

## Discussion

In the present study, we identified a > 600 bp long DMR consisting of at least 24 CpGs, of which we measured 10 CpG sites in the young cohort and 3 CpG sites in the adult cohort. In both cohorts, the DMR had a clearly bimodal methylation pattern. The only gene located in the DMR is the non-coding RNA *nc886* (also called *VTRNA2-1*), which is almost ubiquitously transcribed by RNA polymerase III into a cytosolic ncRNA of 101 nucleotides [8, 26]. Functionally, *nc886* is best characterized by its ability to bind and inhibit Protein Kinase RNA-activated (PKR) [27]. Notably, PKR is a protein kinase activated by double stranded RNA, typically present during viral infections, to negatively regulate ribosomal protein synthesis. PKR activity may also be activated during other inflammatory conditions and by metabolic stress induced by obesity [28]. Moreover, *nc886* is centrally located within the DMR, with its upstream promoter being overlapped by the CpG island [8] (Fig. 1).

It has previously been shown in a Gambian infant cohort that the likelihood of the *nc886* DMR being hypomethylated in blood and hair follicles was significantly increased, when conception occurred during the dry season compared to during the rainy season [4]. Those results indicated that the environmental effect on the *nc886* DMR occurs in the early embryo and that *nc886* is likely to hold capacity as a metastable epiallele, responsive to the fetal nutritional state [4]. Interestingly, *nc886* is the only amongst the ∼100 known imprinted human genes for which polymorphic imprinting has been demonstrated in the population, meaning that it shows mono-allelic methylation in potentially 75% of humans worldwide, whereas the remaining 25% of the population has biallelic hypomethylation [24, 29]. In support, a recent study found that offspring *nc886* methylation in blood cells was associated with the mother's age and socioeconomic status, but not with the offspring’s own genotype, and that the methylation degree of *nc886* in blood was stable for at least 25 years [25]. Interestingly, this study also observed associations between *nc886* methylation degree and glucose and insulin levels during adolescence but not in adulthood, and found that *nc886* expression levels associated positively with insulin levels in early adulthood [25]. Here, we report high conservation of *nc886* hypo- or hemi DNA methylation degree across three human primary tissues, which supports that *nc886* is a metastable epiallele affected by maternal factors present at the time of conception.. We show that young offspring exposed to GDM and maternal obesity in pregnancy display hypomethylation at the *nc886* DMR region, compared to children of control mothers. We further show that this pattern could not be replicated in an older offspring cohort exposed to either maternal GDM or T1D during pregnancy. We can hypothesize that the lack of replication in the older cohort is linked to multiple environmental exposures across time, including diet, physical activity and aging contributing to diminishing epigenetic features established prenatally. In addition, the adult cohort was also characterized by their mothers being significantly leaner, than in the younger cohort, and maternal body weight plays a role in the pathophysiology of GDM. Noteworthy, the adult offspring included were from the second follow-up examination of this cohort and we observed a significant loss to follow-up of particularly the participants diagnosed with either prediabetes or type 2 diabetes in the first follow up study [13]; hence, the participants in the current study were among the healthiest in the cohort, which could contribute to diminishing the observed differences between groups.

### Relationships between genetic and environmental factors and nc886 methylation or expression

It was previously shown that maternal transmission of the A-allele of a C/A SNP (rs2346018) located in a CTCF binding site on the telomeric side of the *nc886* DMR was associated with increased DNA methylation of the *nc886* DMR, and additionally that the probability of DNA methylation at this locus was increased by conception time (rainy season), increased maternal age and decreased offspring childhood BMI [8]. However, other studies were unable to replicate the relationship between genotype at this SNP and probability of DNA methylation [4, 24, 25, 30], which is concordant with findings in the current study: We found that the C/A SNP located within the nc886 gene was not linked to methylation degree in the adult cohort in the tested tissues. A limitation to our study, and the other follow studies [24, 25, 30], is that the parental transmission of alleles was not assessed, and for comparisons of C/C vs. A/A genotypes in our study, the sample sizes are too small to form reliable statistical inferences. Thus, in the future, follow-up studies should focus on specifically assessing the impact of parental transmission of the rs2346018 A-allele and the *nc886* haplotype and on assessing this relationship in larger cohorts.

Environmental exposures have previously been shown to alter methylation levels of the *nc886 DMR:* Maternal age is associated with an increased probability of offspring *nc886* DNA methylation in some studies [24, 25], though not all [25, 30], while the degree of methylation of an individuals blood cells increases as a function of age in some models (though not all), (Additional file 1: Table S9) [31]. It was previously determined that the DNA methylation marks of the *nc886* DMR were stable between tissues and over long periods of time (10–25 years) [25]. In our study, groups of offspring (O-GDM, O-T1D and O-BP in the adult cohort, and O-GDM and O-BP in the young cohort) were well matched for age, and we did not detect any associations between the *nc886 DMR* and age, neither with the maternal age. Environmental contaminants (benzophenone-3) increased *nc886 DMR* methylation in placenta [32], while alcohol consumption alone and in conjunction with smoking decreased *nc886 DMR* methylation in blood [24], while studies in the HELIX cohort did not identify significant associations between *nc886* DNA methylation and early life environmental exposures (tobacco smoking, environmental contaminants or air quality) (Additional file 1: Table S9) [33, 34]. Unfortunately, in the current study we did not have access to maternal smoking and alcohol consumption at the individual level, which have previously been shown to change DNA methylation [35, 36]. In the Danish National Birth Cohort, we the group of GDM mothers have a slightly lower frequency of ‘never smokers’: GDM: 49.3% versus Non-GDM: 53.2% [37], while for the Copenhagen Offspring Cohort of adult offspring, the frequencies of current smokers were O-GDM 46% (77/168), Control 42% (59/141) and O-T1D 36% (57/160)[12], but also for this study we did not have individual level data. Interestingly, increased expression of the DNA repair gene DNAJB6 and increased circulating levels of the DNA repair protein, aprataxin (APTX), were positively correlated with *nc886* methylation (Additional file 1: Table S9) [38]. In relation to GDM, Howe et al. (2020) searched for DMRs in cord blood of newborns of mothers with GDM, but did not identify the *nc886* region as a significantly DMR in their analysis [2]. Of note, Howe et al. (2020) adjusted for a number of maternal covariates, including maternal prepregnancy BMI, whereas the current study did not, which may explain part of the differences in study findings. Taken together, methylation of the *nc886* DMR seems to be associated with both maternal haplotype and pre- or perinatal environment. Longitudinal studies investigating the effect of maternal GDM on *nc886* methylation levels would be interesting, given that this locus is imprinted with a reportedly stable degree of DNA methylation across tissues and time, but time- or postnatal environmental exposures affecting methylation were also demonstrated.

*Nc886* expression levels in SAT in offspring were associated with maternal GDM but not maternal T1D. The severity of intrauterine exposure to hyperglycemia is believed to differ since the O-GDM group comprises offspring of women with diet-treated GDM where maternal glucose levels are mildly elevated, especially in the last part of pregnancy, in contrast to the O-T1D group where maternal hyperglycemia is elevated preconceptionally and mostly at markedly higher glycaemic levels than seen with diet treated GDM. This is supported by the fact that a larger proportion of the O-T1D was born large for gestational age compared to GDM offspring [13]. It is therefore likely that other factors besides specifically intrauterine exposure to hyperglycemia influence the *nc886* expression levels in the offspring. Our results show that maternal pre-pregnancy BMI is, in part, a determining factor of the *nc886* expression levels in SAT of O-GDM; therefore, we can speculate of an interaction between hyperglycemia and obesity in determining *nc866* RNA expression.

### Pre-pregnancy maternal BMI may be a driver of offspring SAT nc886 expression

The methylation pattern of the *nc886* DMR was clearly bimodal in the young offspring cohort (aged 9–16 years) compared with the adult offspring cohort (aged 28–33 years), for which two explanations could be considered: The methylation pattern of the *nc886* DMR could decrease with age or the imprinting periconceptional environment (maternal factors) could be different between the two cohorts. Repeated longitudinal analysis was not possible in our cohorts, but this has been investigated by Silver et al. [4] and Marttila et al. [25], who showed high individual stability of the DNA-methylation marks at this locus when tested 10 or 25 years apart, although another study identified several *nc886* localized CpG sites to be associated with age-dependent changes in DNA-methylation (Additional file 1: Table S9) [31]. In support of altered periconceptional environment between these two cohorts is the observation that mothers of the young cohort were 2 kg/m^2^ heavier than mothers belonging to the adult cohort (Table 1). Moreover, maternal pre-pregnancy BMI was independently associated with the SAT *nc886* expression. However, GDM status remained also significantly associated with offspring SAT *nc886* expression, after adjusting for maternal pre-pregnancy BMI, but to a lesser degree than maternal obesity. Hence, our results indeed support that maternal pre-pregnancy BMI is in part a determining factor of the *nc886* expression levels in SAT of adult offspring of GDM mothers. Importantly, the offspring’s own adiposity in terms of the total body fat percentage, did not influence this association, which suggest that the expression levels are not linked to genetic factors of obesity inheritance. Further support that maternal pre-pregnancy BMI, rather than maternal hyperglycemia alone, could be a primary determinant of the adult offspring SAT *nc886* expression, stems from the observation that the O-T1D participants, who theoretically should have been exposed to hyperglycemia for a longer duration, and to a higher extent than O-GDM, did not have higher expression levels of *nc886* than O-BP participants. Hence, it is possible that an interaction between hyperglycemia and obesity in pregnancy is linked to epigenetic changes of *nc886* in O-GDM and therefore not O-T1D. The fact that maternal pre-pregnancy BMI may be a determinant of *nc886* epigenetic programming supports previous studies showing that lifestyle changes during pregnancy are not very effective, which has led to the hypothesis that interventions in the pre-conception period are more effective, when it comes to preventing GDM and obesity in pregnancy [39]. Today, only few studies initiated weight loss interventions prior to conception [40] including the ‘Pre-Babe’ pilot study that showed a greater reduction in weight loss and higher chance of achieving pregnancy in the meal-replacement group compared to dietary advice [41] and the ‘FIT-PLESE-study’, where a preconception lifestyle intervention (physical activity, meal replacement and medication) targeting weight loss did not improve birth outcome or fertility compared with an exercise intervention not targeting weight loss [42]. However, none of the studies have addressed the impact of a lifestyle intervention before pregnancy on childhood obesity.

It is noteworthy that in the previous EWAS analysis of the younger offspring, the majority of differentially methylated single CpG sites in GDM offspring were found primarily linked to maternal pre-pregnancy BMI [6], which further emphasizes the role of maternal obesity in programming of epigenetic traits. For future studies, it would be beneficial to perform repeated prospective follow-ups of offspring (into adulthood) from mothers with detailed continuous glycaemia measurements during pregnancy to elucidate the relationship between offspring adipose tissue *nc886* and maternal glucose intolerance versus obesity in pregnancy.

### Regulation of nc886 expression by nc886 DNA methylation

Although SAT CpG DNA methylation levels at the nc886 locus were not different between O-BP and O-GDM in adults, the SAT *nc886* RNA levels were markedly different between these groups. It is possible that expression levels of *nc886* are determined by the degree of methylation at sites outside the DMR, however we did not detect single CpG sites in this region that were associated with GDM in the younger cohort [6]. Yet, our results, showing that DMR CpG sites were highly negatively correlated with *nc886* expression levels, are in line with one previous study showing that in vitro methylation of the CpG island of *nc886* suppressed the *nc886* expression compared to the unmethylated control [43]. Therefore, it seems likely that the DMR does regulate the *nc886* levels, but that the differences in DMR methylation degree imposed by GDM, as observed in the young cohort, wanes over time. Interestingly, a recent study investigated epigenome-wide blood DNA methylation patterns across ages 0–20 years, and among the findings of age-dependent methylation variation, were several of the *nc886* DMR CpGs that we identified [31]. However, further studies are needed to establish a clear time- or aging-dependent degree of methylation at this locus. To that extent our data show that the methylation degree in adult offspring is in all- three tissues not as clearly separated to a hypomethylated and hemimethylated group, as seen in blood in the younger offspring. Especially in the muscle tissue the bimodal variation of methylation degree was not as clearly separated as seen in the blood and adipose tissue (Fig. 3C). This is also represented by the correlation between the *nc886* muscle expression and methylation being weaker than in SAT.

### Associations between nc886 expression and offspring metabolic phenotypes

SAT *nc886* expression was positively correlated with parameters of hyperinsulinemia in addition to being negatively correlated with HDL cholesterol in the offspring. Hyperinsulinemia and low levels of HDL are conditions related to the metabolic syndrome and cardio-metabolic diseases. Noteworthy is that no associations at all were identified between skeletal muscle *nc886* levels and phenotype characteristics of the offspring, suggesting that the physiological effect of *nc886* could be more important in adipose tissue than in skeletal muscle. However, studies in experimental models would be required to determine this.

Although the functional actions of *nc886* are not completely characterized, *nc886* has been shown to prevent dsRNA-induced activation of PKR, and instead directing PKR toward the nuclear factor (NF)-κB pathway, which in T-lymphocytes leads to their activation and consequently cytokine production [44]. NF-κB has also been demonstrated to regulate central genes involved in inflammation in adipose tissue [45]. The nc886 genomic region is flanked by the transforming growth factor β-induced (*TGFBI*) and SMAD5 genes, which are both implicated in the TGF-β signaling pathway [46], and which seem to share regulatory regions as determined from chromatin interactions (Fig. 1). Interestingly, *TGFBI* is enriched in extracellular vesicles secreted from visceral adipose tissue of obese individuals together with other proteins involved in adipose tissue inflammation. Moreover, it is elevated in plasma of obese individuals with a history of T2D [47]. For future studies, it will be highly interesting to experimentally characterize *nc886* in response to general inflammation, and whether *nc886* has an inflammatory role in the adipose tissue, which could influence the metabolic health of the offspring. The adipose tissue has emerged as an important player in programming of metabolic health, due to its high potential for plasticity and adaptation to environmental cues [48, 49]. Offspring from obese mothers show increased hyperplasia and hypertrophy of adipocytes postnatally and are often characterized by increased chronic inflammation and infiltration of immune cells in their adipose tissue [50]. Such chronic inflammation is associated with an increased risk of cardio-metabolic disease [51]. Additionally, results from the first follow-up examination of adult offspring showed that maternal overweight but not hyperglycemia was associated with low-grade inflammation in the offspring [52].

## Conclusions

Young offspring exposed to GDM in utero are more likely to have hypomethylation of the metastable epiallele and non-coding RNA *nc886*. The bimodal methylation pattern is not as clearly prevalent in adult offspring exposed to GDM in utero. Our results indicate that the functional role of *nc886* is related to the adipose tissue and that expression levels are influenced by other factors besides intrauterine exposure to hyperglycemia in isolation. This is supported by *nc886* expression in the offspring being significantly associated with maternal pre-pregnancy BMI. In conclusion, adipose tissue *nc886* regulation could have a role in fetal programming of metabolic disease caused by exposure to GDM*,* which is influenced by maternal overweight. However, the mechanisms underlying this programming remain to be fully elucidated.

### Supplementary Information

**Additional file 1**. **Supplementary tables and figures:** Table S1 to S9, and Figure S1 to S2.

## Data Availability

Anonymized personal data are not subject to data protection legislation by the General Data Protection Regulation (GDPR) in the EU and are therefore allowed to be publicly shared. However, the personal data underlying the results in this paper are not possible to fully anonymize and is therefore covered by Section 10 of the Danish Data Protection Act. When personal data covered by Section 10 of the Data Protection Act (which also applies to pseudonymized information) wish to be passed on with a view to publication in a recognized scientific journal, it requires permission from the Danish Data Protection Authority, cf. Section 10, subsection of the Data Protection Act. 3, No. 3. However, the Danish Data Protection Authority can only approve this sharing if there is an authority to do so in the informed consent from the ethical approval cf. Section 2, subsection 10 of the Danish Committees Act. Such authority was not included in the participant information in the two study cohorts included in the present study. It is, therefore, not possible to share pseudonymized information unrestricted. However, upon request, the first and senior authors may share data that support the findings of this study if the data sharing and transfer applies to the GDPR in Denmark.

## Abbreviations

BG: Blood glucose
BMI: Body mass index
CI: Confidence interval
GDM: Gestational diabetes mellitus
O-BP: Offspring of women from the background population
O-GDM: Offspring of women with GDM
OGTT: Oral glucose tolerance test
O-T1D: Offspring of women with type 1 diabetes
SD: Standard deviation
SAT: Subcutaneous adipose tissue
T1D: Type 1 diabetes mellitus
T2D: Type 2 diabetes mellitus
total BF%: Total body fat percentage
