# Supplementary material for: Epigenetics of the non-coding RNA nc886 across blood, adipose tissue and skeletal muscle in offspring exposed to diabetes in pregnancy
Source: Clin Epigenetics. 2024 May 7;16:61. doi: 10.1186/s13148-024-01673-3 (PMC11077860; doi:10.1186/s13148-024-01673-3)

**Supplementary Tables**

| Supplementary Table 1. Target genes, respective primer sequences and expected product | | |
| --- | --- | --- |
| Target Gene | Primer Sequence | Product length (bp) |
| *nc886* *qPCR* | F - 5’ GCTCAAGCGGTTACCTCCTC 3’ | 93 |
|  | R - 5’ GCATAAAAGGGTCAGTAAGCACC 3’ |  |
| *HPRT qPCR* | F - 5’ TGACCTTGATTTATTTTGCATACC 3’ | 51 |
|  | R - 5’ CGAGCAAGACGTTCAGTCCT 3’ |  |
| *TFIIB qPCR* | F - 5’ GTTCTGTTCCAACCTTTGTCT 3’ | 102 |
|  | R - 5’ TGTGTAGCTGCCATCTGTACTT 3’ |  |
| *nc886* pyrosequencing | F - 5’ AGTATAGAGATGGATAGATAGAAAGT 3’ | 91 |
|  | R - 5’ AAAATTTCAATCCCACACTCCTACCC 3’ |  |
|  | Seq. primer: 5’ AGATGGATAGATAGAAAGTT 3’ |  |

| **Supplementary Table 2. Top 3 differentially methylated regions (DMRs) identified** | | | | | | |  |
| --- | --- | --- | --- | --- | --- | --- | --- |
| **Results** | **Chr. location** | **Genomic location start** | **Genomic location end** | **Associated gene** | **DNA methylation difference (%)** | ***Nominal P-value*** | ***FDR corrected P-value*** |
| 1 | Chr5 | 135415948 | 135416613 | *nc886* | -6.51% | ***0.0229*** | ***0.03*** |
| 2 | Chr1 | 205819088 | 205819609 | *PM20D1* | -3.96% | *0.2137* | *0.26* |
| 3 | Chr15 | 67356310 | 67356942 | *LOC102723493* | -5.40% | *0.5496* | *0.54* |
| DNA methylation difference (%) is represented as in GDM offspring compared to controls. Chr.: chromosome, FDR: false rate discovery. | | | | | | | |

| **Supplementary Table 3. Association between adult offspring *nc886* DNA methylation and maternal diabetes and pre-pregnancy BMI** | | | | | | | |
| --- | --- | --- | --- | --- | --- | --- | --- |
|  | ***Association to group compared to O-BP*** | | | | ***Association to***  ***pre-pregnancy mBMI*** | |  |
|  | **O-GDM** | | **O-T1D** | |  |  |  |
| ***Outcomes:*** | **β (95% CI)** | ***p*-value** | **β (95% CI)** | ***p*-value** | **β (95% CI)** | ***p*-value** |  |
| **Blood DNA methylation** (β-estimate shown as difference in CpG DNA methylation degree (%)) | | | | | | |  |
| *CpG 1* | 4.55 (-0.61, 9.71) | *0.08* | 2.66 (-2.41, 7.73) | *0.30* | -0.45 (-0.93, 0.03) | *0.07* |  |
| *CpG 2* | 3.47 (-0.79, 7.73) | *0.11* | 2.20 (-2.15, 6.54) | *0.32* | -0.26 (-0.66, 0.14) | *0.20* |  |
| *CpG 3* | 5.27 (-0.18, 10.72) | *0.06* | 2.77 (-2.78, 8.31) | *0.33* | -0.38 (-0.89, 0.13) | *0.14* |  |
| **SAT DNA methylation** (β-estimate shown as difference in CpG DNA methylation degree (%)) | | | | | | |  |
| *CpG 1* | 3.46 (-3.15, 10.10) | *0.30* | 1.39 (-5.55, 8.32) | *0.69* | -0.30 (-0.95, 0.34) | *0.35* |  |
| *CpG 2* | 4.14 (-1.60, 9.89) | *0.16* | 0.40 (-5.25, 6.06) | *0.89* | -0.33 (-0.87, 0.21) | *0.23* |  |
| *CpG 3* | 4.31 (-3.00, 11.62) | *0.24* | 0.85 (-6.65, 8.35) | *0.82* | -0.40 (-1.11, 0.31) | *0.27* |  |
| **Muscle DNA methylation** (β-estimate shown as difference in CpG DNA methylation degree (%)) | | | | | |  |  |
| *CpG 1* | 5.69 (-0.30, 11.67) | *0.06* | 3.41 (-1.75, 8.56) | *0.19* | -0.10 (-0.63, 0.43) | *0.71* |  |
| *CpG 2* | 5.08 (-0.32, 10.48) | *0.06* | 3.42 (-1.44, 8.27) | *0.17* | -0.08 (-0.54, 0.40) | *0.75* |  |
| *CpG 3* | 5.65 (-0.96, 12.26) | *0.09* | 4.18 (-1.60, 9.96) | *0.15* | -0.06 (-0.65, 0.54) | *0.85* |  |
| Estimated differences in DNA methylation percentage between exposed groups compared to O-BP are presented as β (95% CI) and *p*-value. Adjusted for maternal diabetes status, pre-pregnancy maternal BMI, offspring *nc886* genotype, and sex. Due to Bonferroni correction for multiple testing, a *p*-value was considered statistically significant when *p*≤0.00556. O-BP: offspring of mothers from the background population, O-GDM: offspring of mothers with gestational diabetes, O-T1D: offspring of mothers with type 1 diabetes, SAT: subcutaneous adipose tissue, mBMI: maternal BMI. | | | | | | |  |

| **Supplementary Table 4. Correlations between adult offspring *nc886* DNA methylation across tissues** | | | | | | |
| --- | --- | --- | --- | --- | --- | --- |
| **Blood DNA methylation** | **SAT DNA methylation** | | | **Muscle DNA methylation** | | |
|  | *CpG 1* | *CpG 2* | *CpG 3* | *CpG 1* | *CpG 2* | *CpG 3* |
| *CpG 1* | 0.59 (*<0.0001*) | 0.59 (*<0.0001*) | 0.57 (*<0.0001*) | 0.36 (*<0.0001*) | 0.34 (*0.0001*) | 0.37 (*<0.0001*) |
| *CpG 2* | 0.60 (*<0.0001*) | 0.59 (*<0.0001*) | 0.58 (*<0.0001*) | 0.33 (*0.0001*) | 0.31 (*0.0003*) | 0.35 (*<0.0001*) |
| *CpG 3* | 0.58 (*<0.0001*) | 0.58 (*<0.0001*) | 0.57 (*<0.0001*) | 0.34 (*<0.0001*) | 0.34 (*0.0001*) | 0.35 (*<0.0001*) |

*r* coefficient (*p* value). The correlation analysis was performed via Spearman rank correlation test. SAT: subcutaneous adipose tissue. *n*=120-128. Due to Bonferroni correction for multiple testing, a *p*-value was considered statistically significant when *p*≤0.00556.

| \| **Supplementary Table 5. Frequencies of nc886 rs2346018** **genotype according to adult offspring diabetes group and nc886 hypo/hemi-methylated group** \| \| \| \| \| \| --- \| --- \| --- \| --- \| --- \| \| **Offspring group** \| Homozygous C/C \| Heterozygous C/A \| Homozygous A/A \| *P-value* \| \| O-BP \| 18 (34.6%) \| 28 (53.9%) \| 6 (11.5%) \| *p=0.68* \| \| O-GDM \| 34 (46.0%) \| 30 (40.5%) \| 10 (13.5%) \| \| O-T1D \| 27 (42.2%) \| 30 (46.9%) \| 7 (10.9%) \| \| *In total (n=190)* \| 79 (41.6%) \| 88 (46.3%) \| 23 (12.1%) \| \| ***nc886* group** \| Homozygous C/C \| Heterozygous C/A \| Homozygous A/A \| *P-value* \| \| Hypo-methylated \| 20 (55.6%) \| 13 (36.1%) \| 3 (8.3%) \| *p*=*0.17* \| \| Hemi-methylated \| 51 (37.5%) \| 66 (48.5%) \| 19 (14.0%) \| \| *In total (n=172)* \| 71 (41.3%) \| 79 (45.9%) \| 22 (12.8%) \| \| Proportions of categorical data are shown as n (%) and were tested using Fishers exact test. \| \| \| \| \| |
| --- | --- | --- | --- | --- | --- | --- | --- | --- | --- | --- | --- | --- | --- | --- | --- | --- | --- | --- | --- | --- | --- | --- | --- | --- | --- | --- | --- | --- | --- | --- | --- | --- | --- | --- | --- | --- | --- | --- | --- | --- | --- | --- | --- | --- | --- | --- | --- | --- | --- | --- |

| **Supplementary Table 6. Correlations between adult offspring SAT nc886 expression and DNA methylation** | | | |
| --- | --- | --- | --- |
|  | ***nc886* DNA methylation** | | |
| **SAT *nc886* expression** | SAT CpG 1 | SAT CpG 2 | SAT CpG 3 |
| O-BP (n=29) | -0.48 (*0.008*) | -0.45 (*0.02*) | -0.47 (*0.01*) |
| O-GDM (n=30) | -0.50 (*0.005*) | -0.50 (*0.005*) | -0.61 (*0.0004*) |
| O-T1D (n=43) | -0.37 (*0.014*) | -0.42 (*0.005*) | -0.35 (*0.02*) |
| groups combined (n=102) | -0.38 (<*0.0001*) | -0.35 (*0.0003*) | -0.38 *(<0.0001*) |
| **Muscle *nc886* expression** | Muscle CpG 1 | Muscle CpG 2 | Muscle CpG 3 |
| O-BP (n=37-38) | -0.45 (*0.005*) | -0.49 (*0.002*) | -0.53 (*0.0007*) |
| O-GDM (n=46) | 0.004 (*0.98*) | 0.07 (*0.64*) | -0.02 (*0.90*) |
| O-T1D (n=53) | -0.39 (*0.004*) | -0.34 (*0.01*) | -0.45 (*0.0008*) |
| groups combined (n=136) | -0.25 (*0.003*) | -0.22 (*0.009*) | -0.30 (*0.0004*) |
| *r* coefficient (*p* value). The correlation analysis was performed via Spearman rank correlation test. O-BP: offspring of mothers from the background population, O-GDM: offspring of mothers with gestational diabetes, O-T1D: offspring of mothers with type 1 diabetes, SAT: subcutaneous adipose tissue. | | | |

| **Supplementary Table 7.** **Association between adult offspring *nc886* SAT expression levels and maternal glycaemia status in pregnancy by binary logistic regression** | | |
| --- | --- | --- |
|  | ***Increased SAT nc886 expression*** | |
|  | **Odds ratio (95% CI)** | ***p*-value** |
| ***O-GDM*** |  |  |
| *Maternal hyperglycemia (GDM)* | 25.6 (6.0-101.0) | ***<0.0001*** |
| ***O-T1D*** |  |  |
| *Maternal hyperglycemia (T1D)* | 2.2 (0.9-5.6) | *0.08* |
| Estimated differences by odds ratios of the association between maternal GDM, or T1D status, relative to the control BP group with SAT *nc886* relative gene expression degree by binary logistic regression, with adjustment for maternal BMI, offspring nc886 genotype and sex. T1D: type 1 diabetes, SAT: subcutaneous adipose tissue. | | |

| **Supplementary Table 8. Correlations between the combined cohort of adult offspring *nc886* expression, in SAT and muscle, and clinical parameters** | | | | | | |
| --- | --- | --- | --- | --- | --- | --- |
|  | **SAT** | | | **Muscle** | | |
|  | *n* | *r* coefficient | *p-value* | *n* | *r* coefficient | *p-value* |
| Maternal pre-pregnancy BMI | 159 | **0.22** | ***0.006*** | 175 | 0.05 | *0.49* |
| Total body fat percentage (%) | 161 | 0.07 | *0.35* | 177 | 0.07 | *0.36* |
| Fasting insulin (pmol/L) | 147 | **0.18** | ***0.026*** | 159 | 0.04 | *0.65* |
| Fasting C-peptide (pmol/L) | 161 | **0.18** | ***0.025*** | 177 | 0.02 | *0.79* |
| HDL (mmol/L) | 161 | **-0.24** | ***0.002*** | 177 | -0.03 | *0.68* |
| LDL (mmol/L) | 161 | 0.09 | *0.27* | 177 | -0.07 | *0.39* |
| Total cholesterol (mmol/L) | 161 | 0.01 | *0.89* | 177 | -0.04 | *0.58* |
| The correlation analysis was performed via Spearman rank correlation test. SAT: subcutaneous adipose tissue. | | | | | | |

**Supplementary Table 9. Associations between nc886 DMR CpG methylation phenotypes in publically available databases.**

**EWAScatalog.org data**

| **CpG site** | **Publication** | **PMID** | **Association phenotype** | **Tissue** | **Cohort or consortium name** |
| --- | --- | --- | --- | --- | --- |
| Cg04481923 | Mulder, RH | 33450751 | Negatively corr w age as fixed effect | Whole blood | Generation R  ALSPAC |
|  | Mulder, RH | 33450751 | Positively corr w age as random effect | Whole blood | Generation R  ALSPAC |
|  | Sharp, G | 33517419 | Positively corr with paternal BMI in females (when corrected for maternal BMI. | Cord blood | PACE |
| Cg06536614 | Mulder, RH | 33450751 | Positively corr w age | Whole blood | Generation R  ALSPAC |
|  | Battram, T | 30602389 | Lower methylation degree in Tissue | Buccal cells and peripheral blood mononuclear cells | GECKO  C3ARE |
| Cg26328633 | Mulder, RH | 33450751 | Positively corr w age as random effect | Whole blood | Generation R  ALSPAC |
|  | Jedynak, P | 34523531 | Methylated in placenta following exposure to Benzophenone-3 | Placenta | EDEN |
| Cg25340688 | Mulder, RH | 33450751 | Positively corr w age as random effect | Whole blood | Generation R  ALSPAC |
|  | Battram, T | 30602389 | Lower methylation degree in Tissue | Buccal cells and peripheral blood mononuclear cells | GECKO  C3ARE |
| Cg26896946 | Mulder, RH | 33450751 | Positively corr w age as random effect | Whole blood | Generation R  ALSPAC |
|  | Battram, T | 30602389 | Lower methylation degree in Tissue | Buccal cells and peripheral blood mononuclear cells | GECKO  C3ARE |
| Cg00124993 | Mulder, RH | 33450751 | Positively corr w age as random effect | Whole blood | Generation R  ALSPAC |
|  | Battram, T | 30602389 | Lower methylation degree in Tissue | Buccal cells and peripheral blood mononuclear cells | GECKO  C3ARE |
| Cg08745965 | Mulder, RH | 33450751 | Negatively corr w age as fixed effect | Whole blood | Generation R  ALSPAC |
|  | Battram, T | 30602389 | Lower methylation degree in Tissue | Buccal cells and peripheral blood mononuclear cells | GECKO  C3ARE |
| Cg16615357 | Mulder, RH | 33450751 | Positively corr w age as random effect | Whole blood | Generation R  ALSPAC |
|  | Battram, T | 30602389 | Lower methylation degree in Tissue | Buccal cells and peripheral blood mononuclear cells | GECKO  C3ARE |
|  | Sharp, G | 33517419 | Positively corr with paternal BMI in females (when corrected for maternal BMI. | Cord blood | PACE |
| Cg18797653 | Mulder, RH | 33450751 | Negatively corr w age as fixed effect | Whole blood | Generation R  ALSPAC |
|  | Mulder, RH | 33450751 | Positively corr w age as random effect | Whole blood | Generation R  ALSPAC |
|  | Battram, T | 30602389 | Lower methylation degree in Tissue | Buccal cells and peripheral blood mononuclear cells | GECKO  C3ARE |
|  | Gadd, DA | 35945220 | Positively corr w circulating APTX protein levels | Whole blood | Generation Scotland |
| Cg18678645 | Mulder, RH | 33450751 | Negatively corr w age as fixed effect | Whole blood | Generation R  ALSPAC |
|  | Mulder, RH | 33450751 | Positively corr w age as random effect | Whole blood | Generation R  ALSPAC |
|  | Sharp, G | 33517419 | Negatively corr with maternal BMI in females (when corrected for paternal BMI. | Cord blood | PACE |
|  | Battram, T | 30602389 | Lower methylation degree in Tissue | Buccal cells and peripheral blood mononuclear cells | GECKO  C3ARE |

**ARIES mQTL Database data (http://www.mqtldb.org/)**

| **CpG site** | **Time point** | **SNP** | **Chr** | **Allele** | **Nearest gene(s)** | **Methylation change** |
| --- | --- | --- | --- | --- | --- | --- |
| Cg04481923 | No associations |  |  |  |  |  |
| Cg06536614 | Middle age | rs138531941 | 4 | C | LINC01085 | Negative methylation change |
|  | Middle age | rs10024326 | 4 | A | LINC01085 | Negative methylation change |
| Cg26328633 | Middle age | rs1108021 | 7 | C | DNAJB6 | Positive methylation change |
|  | Middle age | rs62494682 | 7 | G | DNAJB6 | Positive methylation change |
| Cg25340688 | Middle age | rs12533784 | 7 | T | DNAJB6 | Positive methylation change |
|  | Middle age | rs9364796 | 6 | A | SERAC1 | Positive methylation change |
|  | Middle age | rs4716713 | 7 | G | DNAJB6 | Positive methylation change |
|  | Middle age | rs5888715 | 7 | I | DNAJB6 | Positive methylation change |
|  | Middle age | rs9347076 | 6 | A | SYNJ2-SERAC1 | Positive methylation change |
|  | Middle age | rs9365882 | 6 | G | SYNJ2-SERAC1 | Positive methylation change |
|  | Middle age | rs9295308 | 6 | T | SERAC1 | Positive methylation change |
|  | Middle age | rs4709083 | 6 | G | SERAC1 | Positive methylation change |
|  | Middle age | rs9364805 | 6 | T | SERAC1 | Positive methylation change |
|  | Middle age | rs12197370 | 6 | A | SERAC1 | Positive methylation change |
|  | Middle age | rs1108021 | 7 | C | DNAJB6 | Positive methylation change |
|  | Middle age | rs62494682 | 7 | G | DNAJB6 | Positive methylation change |
|  | Middle age | rs9356376 | 6 | G | SERAC1 | Positive methylation change |
|  | Middle age | rs12536831 | 7 | C | DNAJB6 | Positive methylation change |
|  | Middle age | rs7796643 | 7 | T | DNAJB6 | Positive methylation change |
|  | Middle age | rs10225231 | 7 | C | DNAJB6 | Positive methylation change |
|  | Middle age | rs10255470 | 7 | C | DNAJB6 | Positive methylation change |
|  | Middle age | rs10256102 | 7 | C | DNAJB6 | Positive methylation change |
|  | Middle age | rs12201324 | 6 | T | SYNJ2-SERAC1 | Positive methylation change |
| Cg26896946 | Middle age | rs74367945 | 3 | T | LINC01968 | Positive methylation change |
|  | Middle age | rs62494682 | 7 | G | DNAJB6 | Positive methylation change |
|  | Middle age | rs1108021 | 7 | C | DNAJB6 | Positive methylation change |
| Cg00124993 | Middle age | rs2839518 | 21 | C | UBASH3A | Positive methylation change |
|  | Birth | rs12533439 | 7 | A | Y RNA – FAM220A | Positive methylation change |
| Cg08745965 | No associations |  |  |  |  |  |
| Cg16615357 | Adolescence | rs7909882 | 10 | A | CDH23 | Negative methylation change |
|  | Adolescence | rs7893501 | 10 | T | CDH23 | Negative methylation change |
|  | Adolescence | rs3861031 | 10 | G | CDH23 | Negative methylation change |
|  | Adolescence | rs35790161 | 10 | I | CDH23 | Negative methylation change |
|  | Adolescence | rs7914113 | 10 | C | CDH23 | Negative methylation change |
|  | Adolescence | rs10999869 | 10 | G | CDH23 | Negative methylation change |
|  | Adolescence | rs12533439 | 7 | A | Y RNA – FAM220A | Positive methylation change |
| Cg18797653 | Birth | rs1009069 | 5 | C | WWC1 | Negative methylation change |
| Cg18678645 | No associations |  |  |  |  |  |

PMID: 35945220/DOI: 10.1038/s41467-022-32319-8

**HELIX eQTM database**

**https://helixomics.isglobal.org/**

| **CpG site** | **Pregnancy** | **Childhood** |
| --- | --- | --- |
| Cg04481923 | No association | No association |
| Cg06536614 | No association | No association |
| Cg26328633 | No association | No association |
| Cg25340688 | No association | No association |
| Cg26896946 | No association | No association |
| Cg00124993 | No association | No association |
| Cg08745965 | No association | No association |
| Cg08745965 | No association | No association |
| Cg08745965 | No association | No association |
| Cg16615357 | No association | No association |
| Cg18797653 | No association | No association |
| Cg18678645 | No association | No association |

PMID: 36411288 https://doi.org/10.1038/s41467-022-34422-2

**Supplementary Figures**

**Supplementary Figure 1**: Unsupervised hierarchical clustering analyses showed as dendrograms for **A.** the **young offspring** cohort (n=185), with clustering conducted for the 10 CpGs from blood DNA covered by the array study, and **B.** the **adult offspring** cohort (n=186), with clustering analysis conducted for the three CpGs from blood DNA covered by the specific pyrosequencing assay. The hypo-methylated offspring are marked in each cluster with red.

**Supplementary Figure 2**: **Adult offspring** correlations between nc886 relative expression and **A**. maternal BMI, **B.** fasting insulin, **C**. fasting C-peptide, and **D**. HDL. *r*: Spearman’s rank coefficient. *p*: p-value. HDL: High-density lipoprotein.


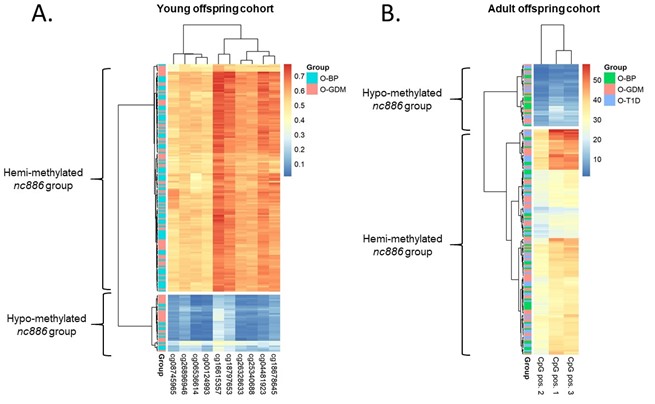


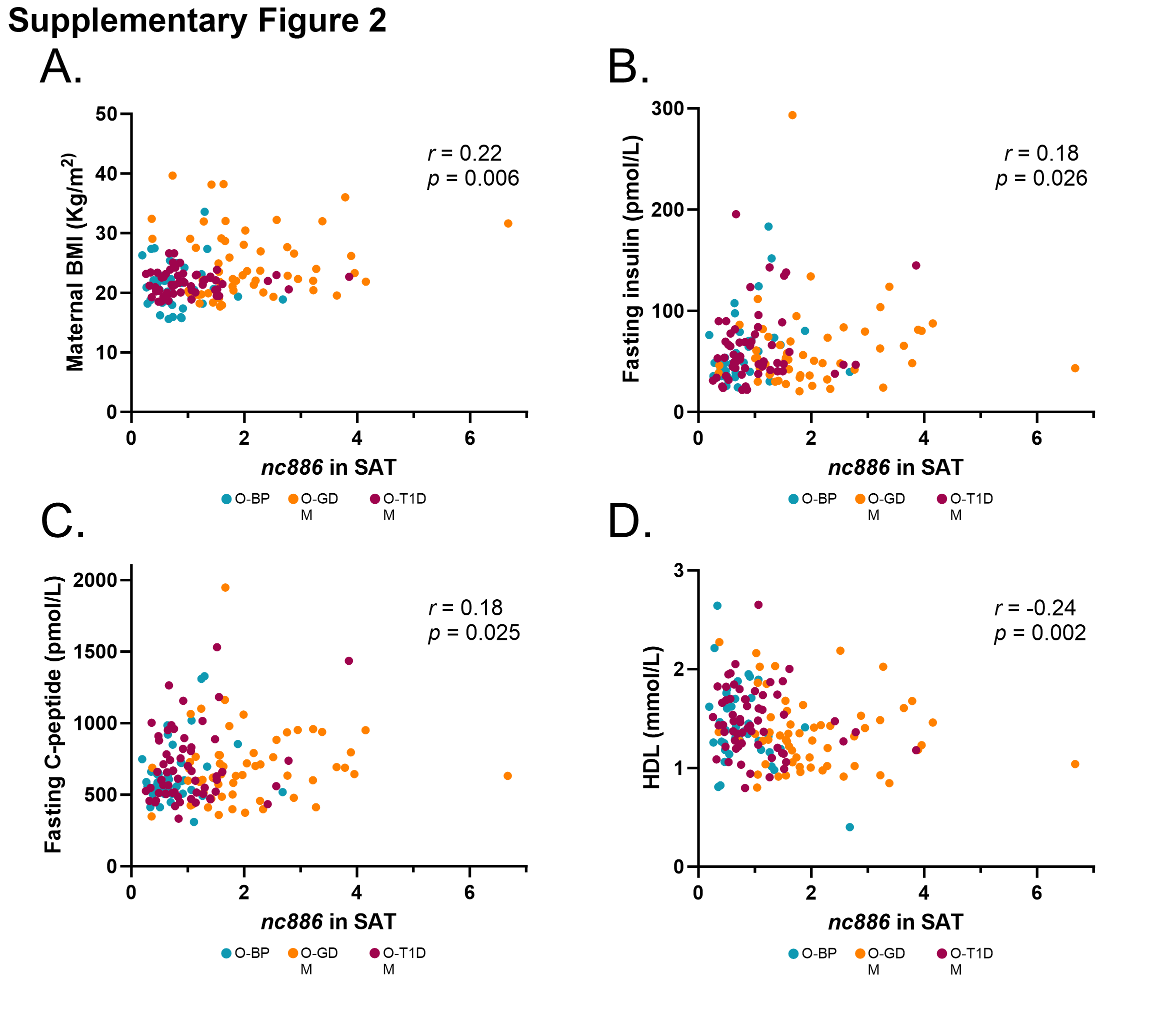

Supplement: Supplementary file 1 — Additional file 1. Supplementary tables and figures: Table S1 to S9, and Figure S1 to S2. [file 13148_2024_1673_MOESM1_ESM.docx]
